# Supplementary material for: Comparison of the TEMPO binocular perimeter and Humphrey field analyzer
Source: Sci Rep. 2023 Dec 1;13:21189. doi: 10.1038/s41598-023-48105-5 (PMC10692178; doi:10.1038/s41598-023-48105-5)
Supplement: Supplementary file 1 — Supplementary Figures. [file 41598_2023_48105_MOESM1_ESM.pdf]

Supplemental Figure 1.
Bland-Altman diagram with median difference and agreement limits (including 95% of all difference values). The solid line represents the line of best fit from linear regression.

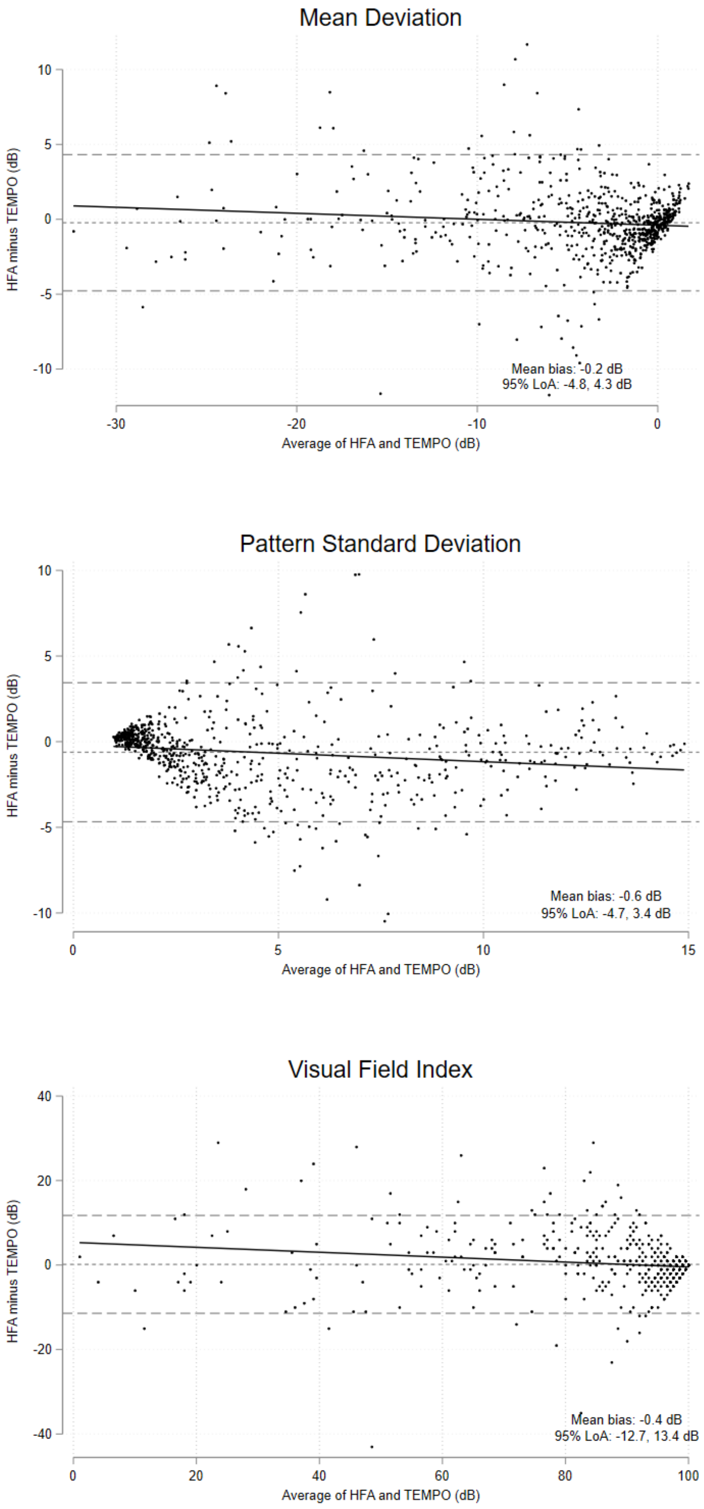

Supplemental Figure 2.

Results of a usability survey comparing TEMPO and Humphrey field analyzer.

(A) Which device do you prefer?

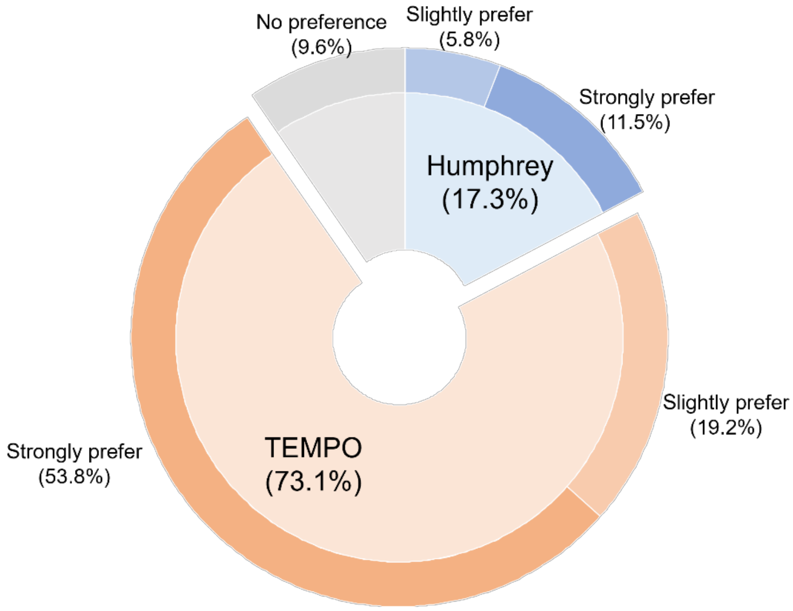

(B) Did you have any difficulty with the simultaneous examination of both eyes using a novel device?

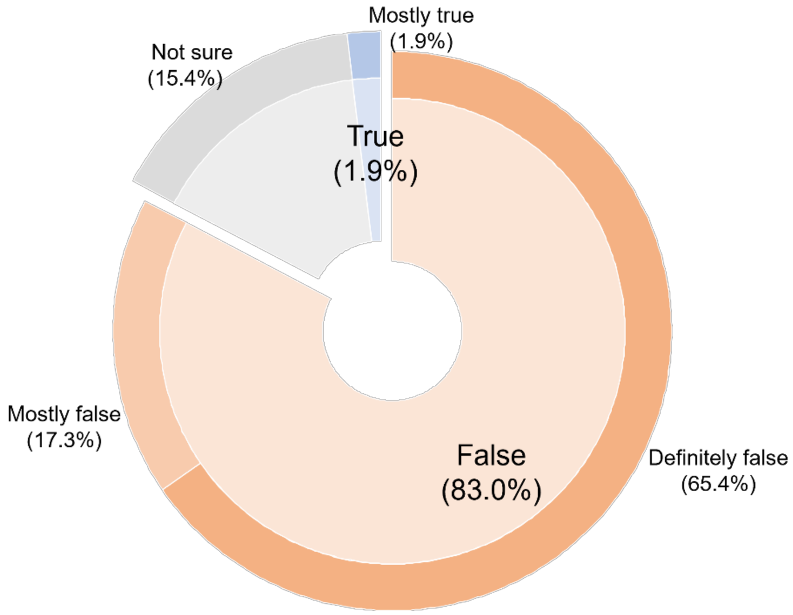

(C) Was the screen easy to see?

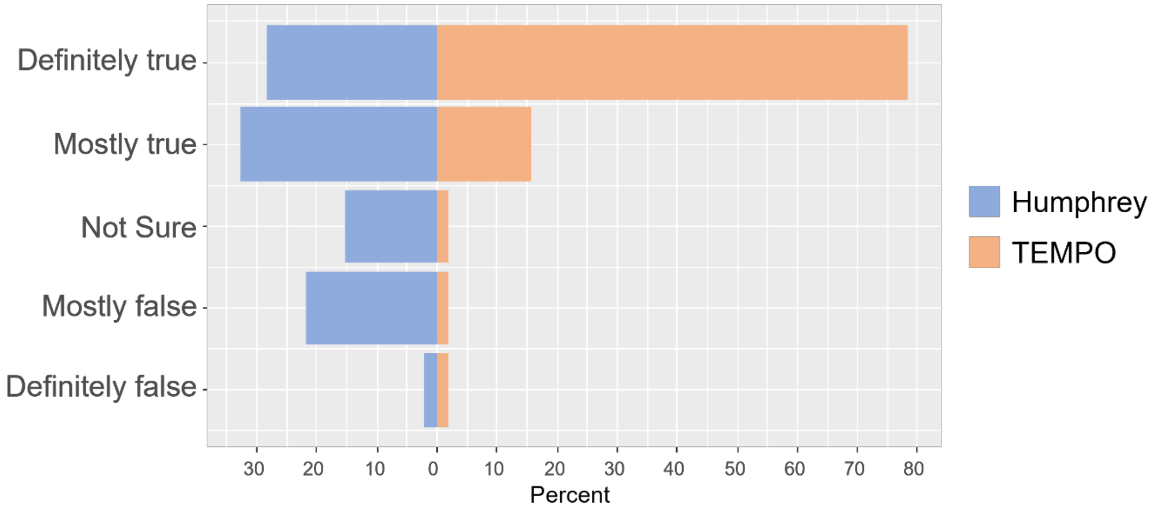

(D) Was it easy to concentrate?

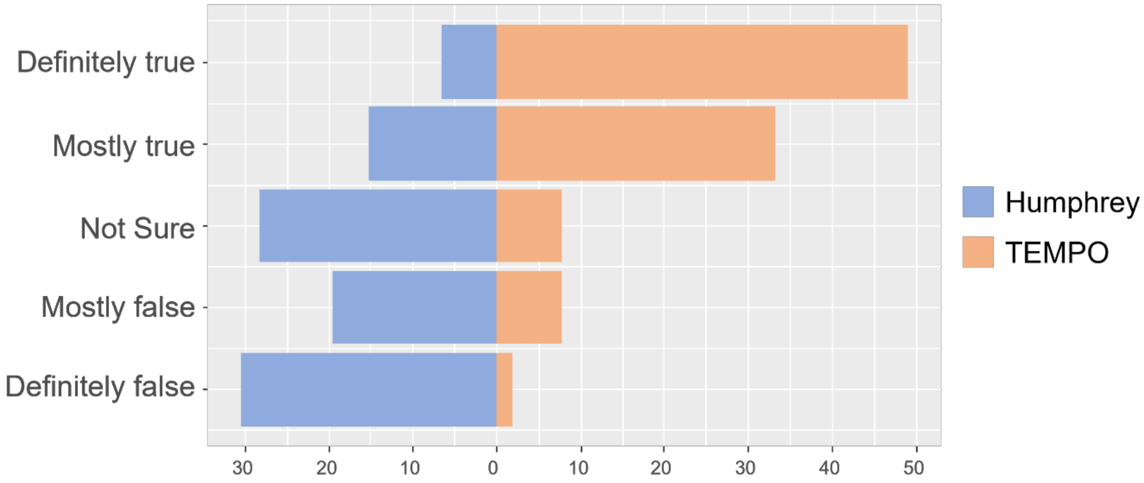

(E) Was the test time short?

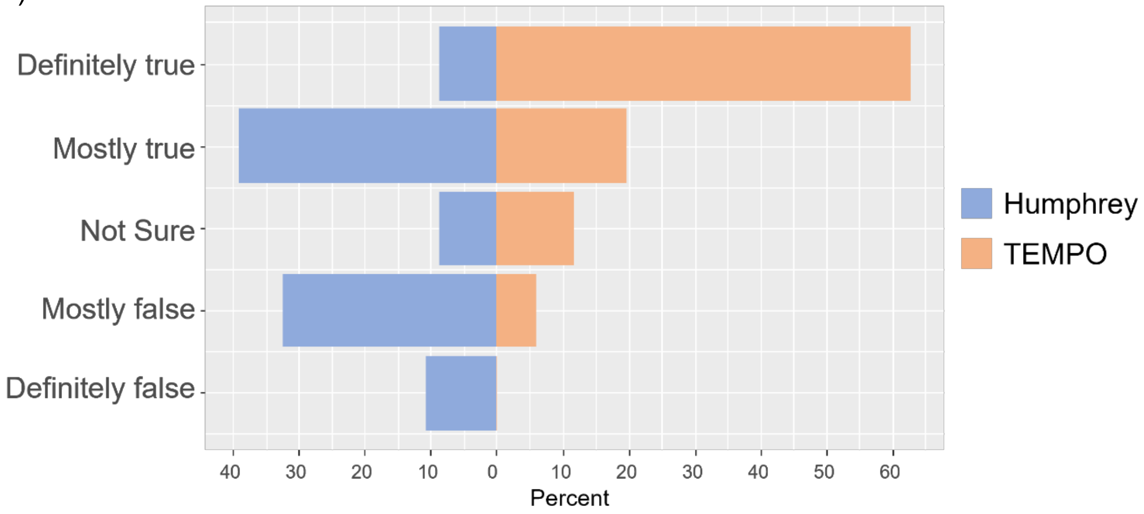

Supplemental Figure 3.

Analysis of sensitivities at each test location between TEMPO and Humphrey Field Analyzer (HFA). All eyes plotted as the right eye.

(A) The graph shows the average differences (TEMPO minus HFA) for total deviation between TEMPO and HFA at each location. A warmer color indicates that the sensitivity for HFA is higher than for TEMPO.

(B) The graph shows the P-values from Wilcoxon signed-rank tests for each location.

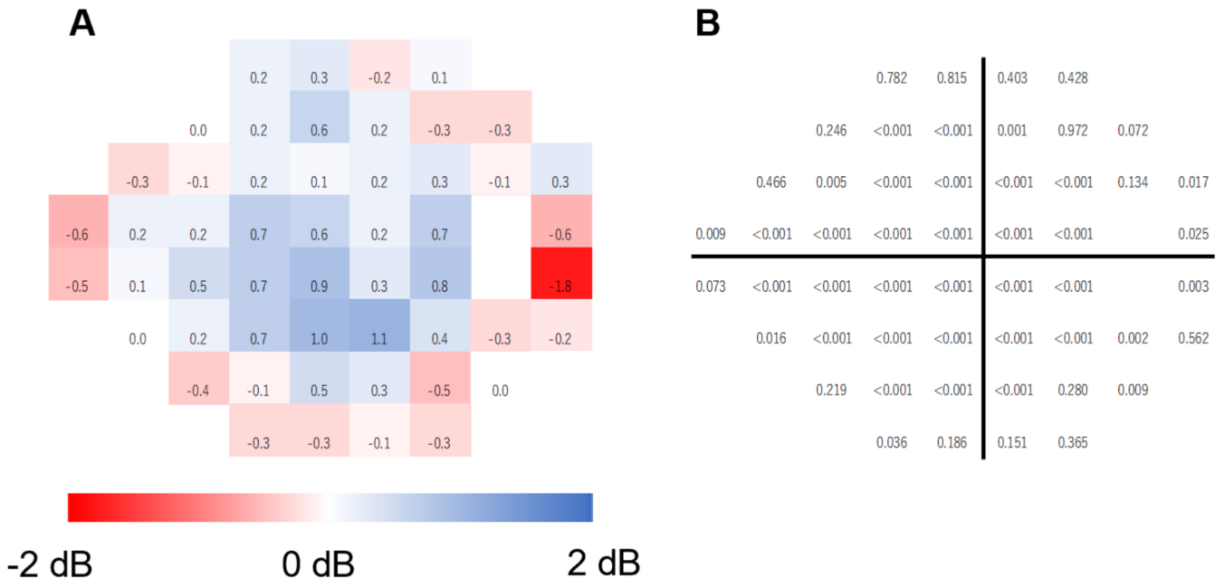

This figure was made using Microsoft Excel (Microsoft Corporation, microsoft.com/en-au/microsoft-365/excel).
